# Supplementary material for: Quantitative real-time analysis of the efflux by the MacAB-TolC tripartite efflux pump clarifies the role of ATP hydrolysis within mechanotransmission mechanism
Source: Commun Biol. 2021 Apr 22;4:493. doi: 10.1038/s42003-021-01997-3 (PMC8062640; doi:10.1038/s42003-021-01997-3)
Supplement: Supplementary file 2 — Supplementary Information [file 42003_2021_1997_MOESM2_ESM.pdf]

Supplementary Material

Quantitative real-time analysis of the efflux by the MacAB-TolC tripartite efflux pump clarifies the role of ATP hydrolysis within mechanotransmission mechanism.

Hager Souabni, William Batista dos Santos, Quentin Cece, Laurent J. Catoire,

Dhenesh Puvanendran, Vassiliy N. Bavro, Martin Picard.

Supplemental Material: Figure S1

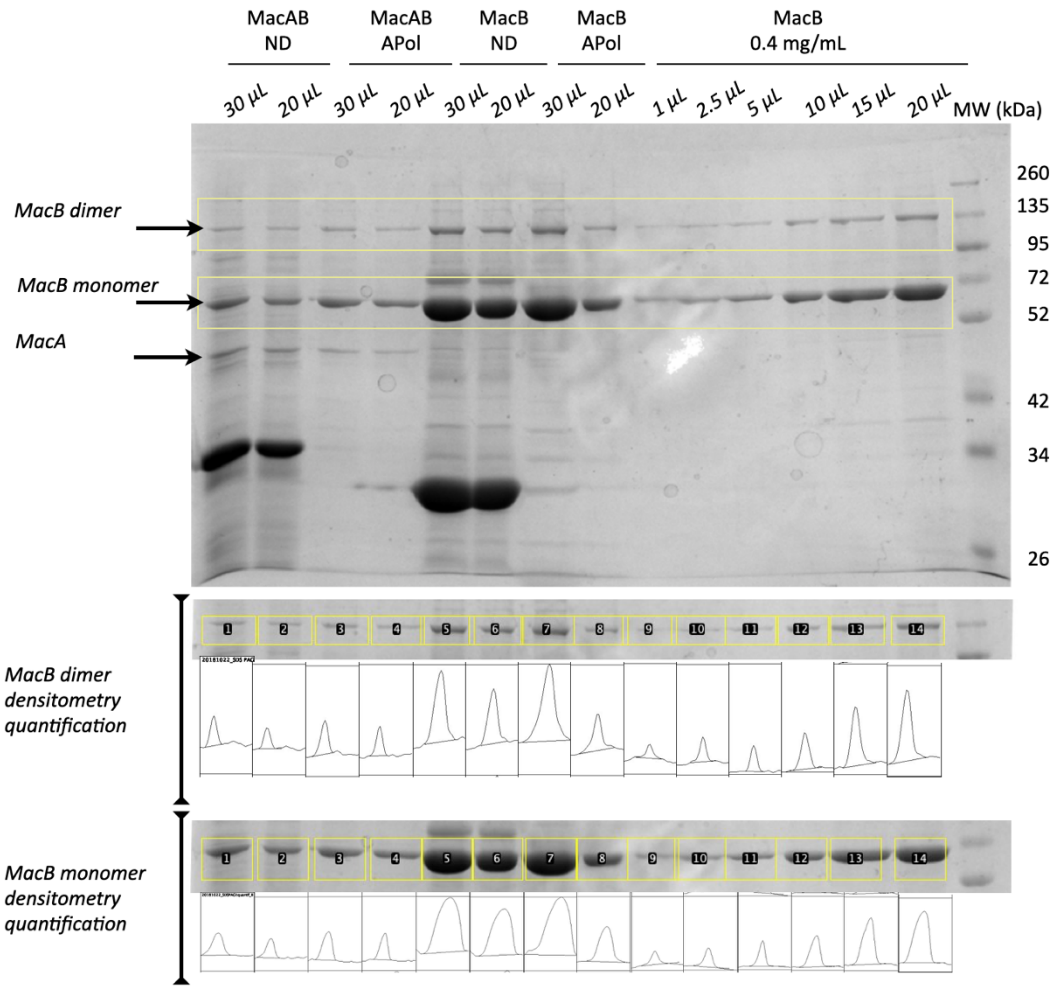

V macB (µL)	µg B	bande dimer	bande monomer	total intensity
1	0,4	587	1360	1947
2,5	1	959	1969	2928
5	2	986	2188	3174
10	4	1801	4087	5888
15	6	3252	6817	10069
20	8	4294	9865	14159

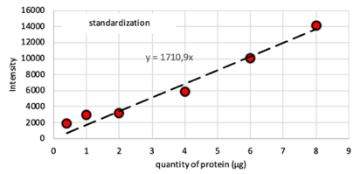

	dimer band intensity	monomer band intensity	total intensity	total intensity / slope	µg/µl	[MacB]total (µg/µL)	total intensity / slope	µg/µl	[MacB]dimer (µg/µL)
30µl ND AB	1178	3362	4 540	2,653574142	0,088452471		0,688526507	0,022950884	
20µl ND AB	809	2123	2 932	1,713717926	0,085685896	0,087069184	0,472850546	0,023642527	0,023296705
30µl AB Apol	1507	3468	4 975	2,90782629	0,096927543		0,880822959	0,029360765	
20µl AB Apol	1101	2582	3 683	2,152668186	0,107633409	0,102280476	0,643520954	0,032176048	0,030768406
30µl ND B	4693	15179	19 872	11,61493951	0,38716465		2,74300076	0,091433359	
20µl ND B	3115	11512	14 627	8,549301537	0,427465077	0,407314864	1,820679175	0,091033959	0,091233659
30µl B Apol	6450	17066	23 516	13,74481267	0,458160422		3,769945643	0,125664855	
20µl B Apol	2039	6220	8 259	4,827283886	0,241364194	0,349762308	1,191770413	0,059588521	0,092626688

SDS-PAGE gel analysis and estimation of MacB quantity after purification and reconstitution into nanodiscs (ND) or amphipols (APol), prior to their use for the ATPase activity assays.
Protein samples were diluted at a 1:1 ratio with Laemmli 2× buffer solution (Bio-rad) with 5% 2-mercaptoethanol (Sigma-Aldrich) as a denaturing agent and without heat denaturation. Protein samples (volumes indicated at the top of each gel) were loaded in the wells and electrophoresis was run at 200 V for 30 min in a Mini-Protean Tetra cell (Bio-rad) using TGX running buffer. Gels were washed with distilled water and stained with Coomassie Blue. and imaged in a Molecular Imager ChemiDoc XRS System (170-8070, Bio-rad) under white light epi-illumination. Images were saved as a TIFF file and analyzed using ImageJ (NIH). The Gel Analyzer tool of ImageJ was used to determine the profiles of each lane of the gel. The overall MacB protein load (monomer + dimer) or the proportion corresponding to MacB dimer only was correlated with the corresponding peak area in the densitometry profile (shown below each band selection, depicted by yellow boxes), shown at the bottom of the figure (standardization calculated in Excel spreadsheet).

Supplemental Material: Figure S2

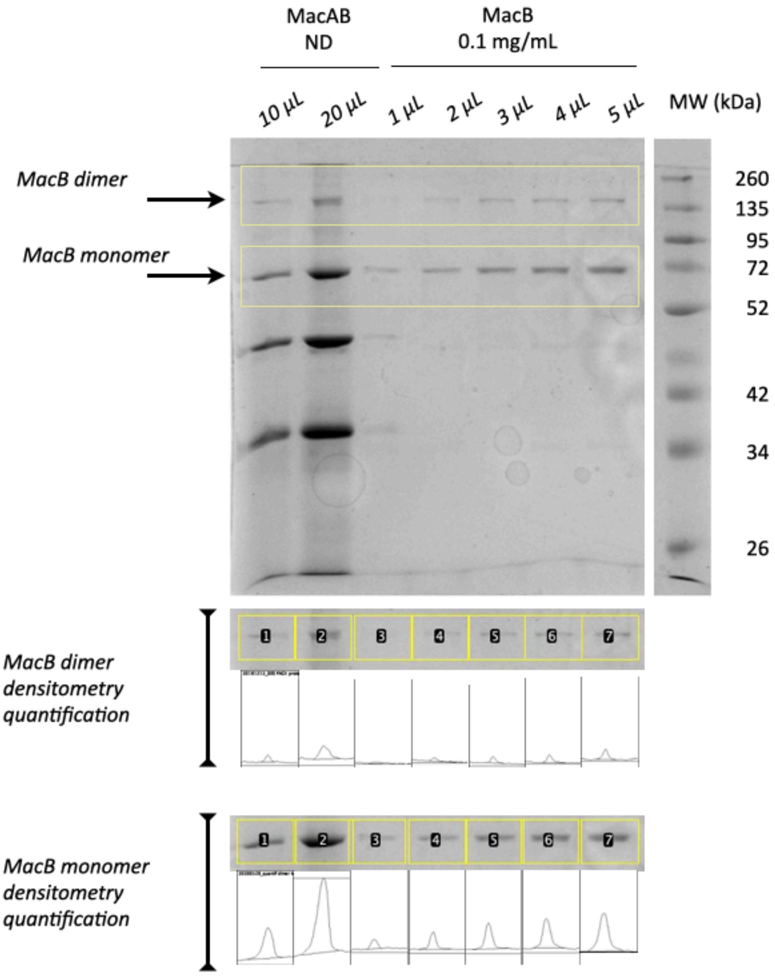

V MacB (µL)	µg B	bande dimer	band monomer	total intensity
1	0,1	194	564	758
2	0,2	164	1137	1 301
3	0,3	283	1818	2 101
4	0,4	381	2290	2 671
5	0,5	522	3523	4 045

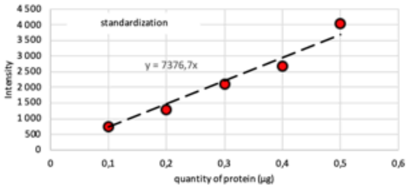

	dimer bande intensity	monomer bande intensity	total intensity	total intensity / slope	µg/µL	[MacB]total
10µl ND AB	298	2882	3180	0,431087071	0,043108707	
20µl ND AB	1173	7826	8999	1,219922187	0,060996109	0,052052408

SDS-PAGE gel analysis and estimation of MacB quantity after purification and reconstitution into nanodiscs (ND), prior to their use for the roxithromycin transport assays.
Protein samples were diluted at a 1:1 ratio with Laemmli 2× buffer solution (Bio-rad) with 5% 2-mercaptoethanol (Sigma-Aldrich) as a denaturing agent and without heat denaturation. Protein samples (volumes indicated at the top of each gel) were loaded in the wells and electrophoresis was run at 200 V for 30 min in a Mini-Protean Tetra cell (Bio-rad) using TGX running buffer. Gels were washed with distilled water and stained with Coomassie Blue. and imaged in a Molecular Imager ChemiDoc XRS System (170-8070, Bio-rad) under white light epi-illumination. Images were saved as a TIFF file and analyzed using ImageJ (NIH). The Gel Analyzer tool of ImageJ was used to determine the profiles of each lane of the gel. The overall MacB protein load (monomer + dimer) was correlated with the corresponding peak area in the densitometry profile (shown below each band selection, depicted by yellow boxes), shown at the bottom of the figure (standardization calculated in Excel spreadsheet).

Supplemental Material: Figure S3

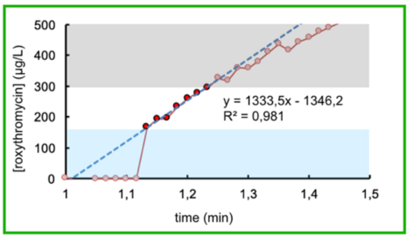

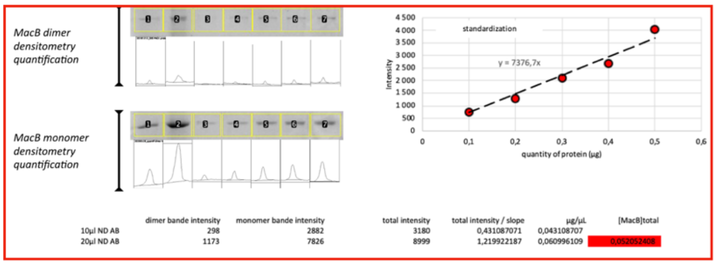

V = 1333.5 µg . ml^{-1}. min^{-1}

= 1.59 µmol . ml^{-1}. min^{-1} in the presence of [MacB] = 0.052 µg . µl^{-1} (volume proteoliposome = 500µL => qty (MacB) = 26 µg)

= 61.15 nmol . µL^{-1}. mg^{-1} . min^{-1}

Aqueous trapped volume for liposomes prepared after extrusion through membrane of increasing pore size and corresponding rate of transport :

pore size	aqueous trapped volume (µL/µmol lipid)	aqueous trapped volume (µL)	rate of transport (nmol Rox . mg^{-1} . min^{-1})
30 nm	1 µL/µmol	0.665	41
**100 nm**	**1.6 µL/µmol**	**1.064**	**65**
200 nm	2.1 µL/µmol	1.396	85
400 nm	3.5 µL/µmol	2.3275	142

[lipid] in the liposome suspension = 1.33 mM
(Bartlett titration)

Table adapted from Mayer, L.D., M.J. Hope, and P.R. Cullis 1986
Vesicles of Variable Sizes Produced by a Rapid Extrusion Procedure.
Biochimica et Biophysica Acta (BBA) - Biomembranes 858(1): 161–168.)

Details of the calculation.

From the kinetics of roxithromycin transport (green panel reproduced from the experiment shown Figure 2b), we estimate a rate of steady-state roxithromycin transport of 1335.5 µg.ml^{-1}.min^{-1}, corresponding to 1.59 µmol.ml^{-1}.min^{-1} (MW roxithromycin = 837 Da / cuvette volume = 520µL). From the densitometry measurements (red panel reproduced from the experiment shown Supplementary Figure S2), we estimate that this rate of transport was obtained from a quantity of 26 µg MacB in nanodiscs. Hence, we estimate a rate of 61.15 nmol of roxithromycin transported per mg of MacB, per minute in a volume of 1µL. Note that a 20% error on the estimation of the protein concentration (0,052 ± 0,009 mg/ml) would lead to a transport value of 61.15 ± 11 nmol.µL^{-1}.mg^{-1}.min^{-1}.
[1]

We evaluate the internal volume of the liposome by comparison with the work of Mayer and collaborators where they experimentally measured the aqueous trapped volume by titration of entrapped Na^{22} or [^{14}C]inulin for liposomes prepared after extrusion through polycarbonate filters of increasing pore diameter. For liposomes prepared after extrusion through 100nm membranes, *i.e* in conditions similar to ours, they calculated a volume of 1.6µL per µmol lipid used for the liposome preparation. We calculated the lipid concentration by Bartlett titration and found a value of 1.33 mM. Hence we extrapolate that the aqueous trapped volume in our suspension is 1.064 µL.
[2]

Note that we reach a similar trapped volume value using Nanoparticle Tracking Analysis (NTA), a technique that uses particle-by-particle light scattering to provide size information based on their Brownian motion. By contrast to classical dynamic light scattering (DLS), each particle is sized independently, measured simultaneously and NTA's sizing principle is absolute. The readout of NTA analysis consists in the absolute number of particles of a given diameter over a range of statistical intervals from 5 nm – 1000 nm. By extrapolating the theoretical entrapped volume for each particle size interval and multiplying the latter by the corresponding number of (measured) particle we can calculate the value of the overall internal trapped volume: we end up with a value of 1.05µL.

From the above ([1] + [2]), we conclude that the rate of roxithromycin transport is 65 nmol per nmol MacB and per minute.

Supplemental Material: Figure S4

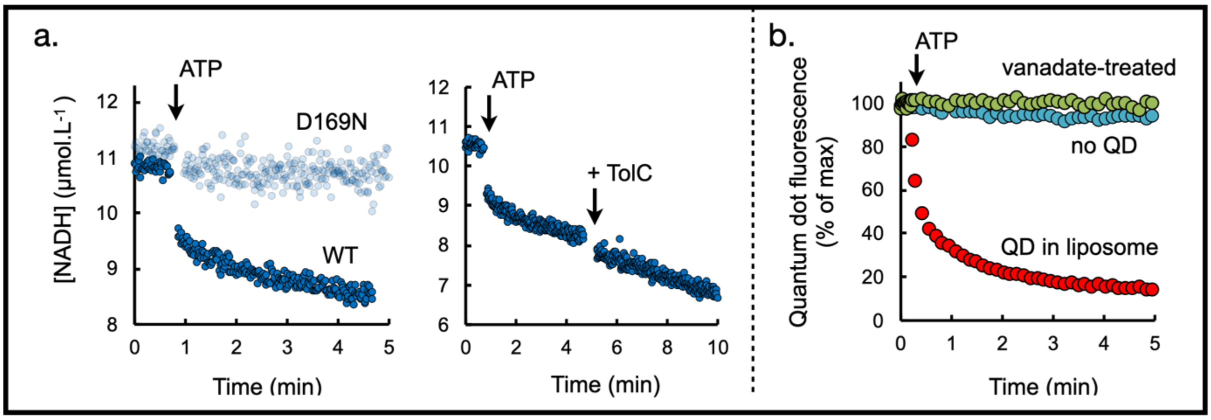

a) Variation of the NADH concentration as a function of time after 10-fold dilution of the MacAB nanodiscs (prepared from WT versus mutant MacB (left) and WT in the absence or in the presence of TolC; *c.a* 10 µg MacB, as standardized by SDS PAGE densitometry, see supplemental Figure S1) into 20 mM Tris pH 8, 50 mM NaCl, 2mM $MgCl_2$ containing the coupled-enzyme assay (see Methods for details) and addition of 1 mM ATP (represented by the arrow). The proportionality coefficient between NADH fluorescence and the NADH concentration allowed the conversion of the fluorescence changes (not shown here) into variations in NADH concentration. Changes due to dilution have been corrected for. NADH fluorescence was measured with an excitation wavelength set at 350 nm and emission at 460 nm.

b) CdTe quantum dot fluorescence is measured as a function of time after 10-fold dilution of the MacAB / TolC complex into 20 mM Tris pH 8, 50 mM NaCl, 2mM $MgCl_2$, addition of 7µM roxithromycin and 7µM vanadate (not shown here for the sake of clarity) and of 1 mM ATP (represented by the arrow). Measurements were performed in the presence of MacAB-ND and QD-loaded TolC proteoliposomes (red curve), in the presence of MacAB-ND and QD-free TolC proteoliposomes (blue curve) or in the presence MacAB-ND treated with vanadate and then mixed with QD-loaded TolC proteoliposomes (green curve). Vanadate acts as a potent inhibitor of many ATPases, as it mimics the transition state for the γ-phosphate of ATP during hydrolysis thus stabilizing the transition state conformation. The MacAB nanodiscs (25µg MacB, as standardized by SDS PAGE densitometry, see supplemental Figure S2) and TolC proteoliposomes were preincubated for at least 1h at room temperature prior to their addition in the fluorescence cuvette, roxithromycin was added in the cuvette at least 10 minutes before addition of ATP, vanadate was added 1 minute after roxithromycin. Changes due to dilution have been corrected for. Traces were normalized as 100% right before addition of ATP.
