# Supplementary material for: Quantitative real-time analysis of the efflux by the MacAB-TolC tripartite efflux pump clarifies the role of ATP hydrolysis within mechanotransmission mechanism
Source: Commun Biol. 2021 Apr 22;4:493. doi: 10.1038/s42003-021-01997-3 (PMC8062640; doi:10.1038/s42003-021-01997-3)
Supplement: Supplementary file 3 — Description of Additional Supplementary Files [file 42003_2021_1997_MOESM3_ESM.pdf]

Description of Additional Supplementary Files

File Name: Supplementary Data 1

Description: NTA raw data

File Name: Supplementary Data 2

Description: raw data for Figure 2 ATPase activity

File Name: Supplementary Data 3

Description: raw data for Figure 2 QD Rox transport

File Name: Supplementary Data 4

Description: Raw data for supplementary material Figure 4
